# Strengthened Decellularized Porcine Valves via Polyvinyl Alcohol as a Template Improving Processability

**DOI:** 10.3390/polym16010016

**Published:** 2023-12-20

**Authors:** Qingqing Chen, Chaorong Wang, Han Wang, Jinfeng Xiao, Yingshan Zhou, Shaojin Gu, Weilin Xu, Hongjun Yang

**Affiliations:** 1 College of Materials Science and Engineering, Wuhan Textile University, Wuhan 430200, China; qingqingchen086@163.com (Q.C.); adaswag724@outlook.com (C.W.); 2011020@wtu.edu.cn (Y.Z.); gusj@wtu.edu.cn (S.G.); 2 Key Laboratory of Green Processing and Functional New Textile Materials of Ministry of Education, Wuhan Textile University, Wuhan 430200, Chinastorm383382@outlook.com (J.X.); weilin_xu@wtu.edu.cn (W.X.); 3 Institute for Frontier Materials, Deakin University, Geelong, VIC 3216, Australia

**Keywords:** heart valve, decellularization, immunogenicity, polyvinyl alcohol, processability

## Abstract

The heart valve is crucial for the human body, which directly affects the efficiency of blood transport and the normal functioning of all organs. Generally, decellularization is one method of tissue-engineered heart valve (TEHV), which can deteriorate the mechanical properties and eliminate allograft immunogenicity. In this study, removable polyvinyl alcohol (PVA) is used to encapsulate decellularized porcine heart valves (DHVs) as a dynamic template to improve the processability of DHVs, such as suturing. Mechanical tests show that the strength and elastic modulus of DHVs treated with different concentrations of PVA significantly improve. Without the PVA layer, the valve would shift during suture puncture and not achieve the desired suture result. The in vitro results indicate that decellularized valves treated with PVA can sustain the adhesion and growth of human umbilical vein endothelial cells (HUVECs). All results above show that the DHVs treated with water-soluble PVA have good mechanical properties and cytocompatibility to ensure post-treatment. On this basis, the improved processability of DHV treated with PVA enables a new paradigm for the manufacturing of scaffolds, making it easy to apply.

## 1. Introduction

The heart valve is one of the vital organs that maintain normal blood circulation and prevent regurgitation of blood [1,2]. Heart valve disease occurs at any age and can present as early as birth [3,4,5]. Nearly one-third of the most significant congenital malformations are caused by congenital heart disease (CHD); valve replacement surgery may be necessary for children with severe congenital heart disease during the first few days of life [6,7,8,9]. The calcified or broken heart valve leads to regurgitation of blood and a strike of the blood circulation system. Therefore, heart valve replacement is needed [10]. There are two types of heart valves used in heart valve replacement: mechanical valves and bioprosthetic valves. Every valve replacement has relative benefits and drawbacks; mechanical heart valves, for example, are more durable but require anti-coagulation therapy to prevent blood clots from forming. Meanwhile, the structure and hemodynamics of bioprosthetic valves are biomimetic, and the patient does not need to take anti-coagulation medication [11]. The application prospect of bioprosthetic valves brings great potential in supplying clinical demand.

According to previous reports [12,13,14,15], porcine valves are commonly used in bioprosthetic valves owing to the similar anatomy to humans. Both decellularized porcine and bovine pericardium are acceptable as substitutes for transplantation, but the porcine pericardium has more long-term performance [16]. However, decellularized heart valve replacement remains unsatisfactory due to the lack of mechanical strength. For example, to fulfill the desired properties of the tissue-engineered heart valve (TEHV) for accelerating endothelialization and regeneration of the decellularized valves, Dong et al. prepared a nano-drug controlled release system through encapsulating vascular endothelial growth factor (VEGF) into polycaprolactone (PCL) nanoparticles, then the PCL and decellularized heart valves were mixed to fabricate TEHV using the Michael addition reaction, which not only could guarantee encapsulation rate of VEGF in PCL maintained at 82%, but also could improve mechanical properties of decellularized valves [17]. Owing to the homology of human heart valves, porcine valves are widely used in heart replacement. Generally, there are limitations in the treatment of valve replacements using mechanical and glutaraldehyde (Glut)-treated [18] bioprostheses. Glutaraldehyde-treated heart valves suffer from cytotoxicity, poor hemocompatibility, and calcification [19]. Then, decellularization is extensively used.

Decellularization is a clinically feasible method, mainly by removing the immunogenic cellular components of human or animal organs/tissues to build biomaterial models [20]. Previous reports established that following decellularization, components like collagen, elastin, fibronectin, and laminin were still present and preserved their original physicochemical signatures and biological properties [21,22,23,24,25]. Given the advantages listed above, the technique of decellularization is frequently seen in the fabrication of bioprosthetic valves. By decellularizing the xenogeneic heart valves, Katja Findeisen et al. were able to remove the alloantigen for the purpose of reducing immunogenicity while keeping the original matrix and properties [26]. Despite the fact that decellularization can preserve biocompatibility while eliminating immunogenicity, the single valve is extremely tiny and soft in texture, thus limiting its processability, which becomes more flexible and has less strength after decellularization. Nevertheless, biomechanical properties are one of the necessary factors to be considered in the preparation of bioprosthetic valves [27].

Processability is the first tricky issue for the applications of decellularized valves. As a water-soluble polymer, polyvinyl alcohol (PVA) has good biocompatibility [28]. Compared with other alternating polymers, the low molecular weight of PVA can be dissolved at room temperature. Further, the PVA possesses unique mechanical properties, which may act as a dynamic template to construct advanced TEHV. For example, MARIA TERESA CONCONI et al. [29] used PVA as internal concentric layers on a decellularized vascular matrix, which proved that composite grafts can promote the growth of endothelial cells, indicating the compatibility of PVA. Based on prior research [30,31], a key limitation of previous developments is the restricted ability to achieve a secure grip and high-resolution tip design due to the natural softness and small size of the decellularized valves. This underscores the urgent need for a safe and convenient method to enhance the processability of decellularized valves, enabling long-term and effective processing. To address this limitation, the purpose of PVA fixation is to enhance suture possibilities, particularly in fiber-reinforced processes. Oriented fibers can regulate the behavior of cells while enhancing the mechanical properties of DHVs and promoting valve reconstruction. Unfortunately, the small size and great flexibility of the valve make it difficult to construct a fiber-woven structure on the decellularized valves by suture with traditional frame fixation. Without the presence of a PVA layer, the valves would shift during suture puncture, preventing the desired suture result. PVA is chosen as a template material to ensure the valves undergo processing without incurring damage. Hence, adding a removable PVA template to impart processability to the decellularized valve was considered, which means that the decellularized valves can be post-processed for subsequent use, making it easy to apply.

**Figure 1:**
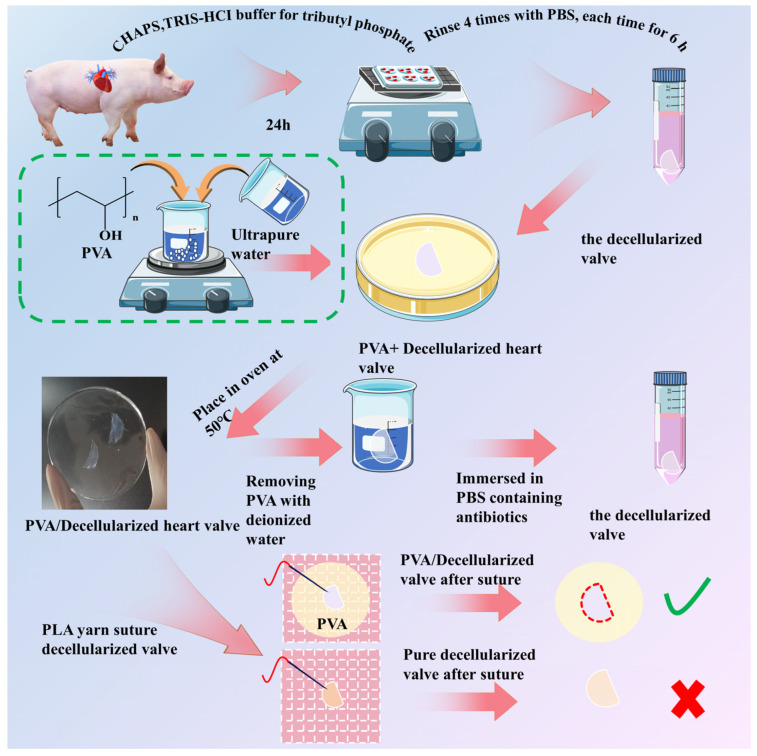
Schematic diagram of PVA/decellularized heart valves. The bottom of the picture is a schematic diagram of the PVA/decellularized heart valve suture.

To address the problem of softening valves after decellularization, in this study, PVA was adopted as an auxiliary material to strengthen decellularized porcine valves (DHVs) to improve processability while maintaining the original properties. The PVA/decellularized porcine valves (PDV) composites were obtained using the template method (Figure 1). The optimal concentration of PVA was investigated to strengthen decellularized porcine valves. Then, the PVA of PDV composites was subsequently removed using the water-soluble method, and the physical and biological properties of the samples before and after treatment were systematically compared. The mechanical test showed that DHVs treated with PVA solution affected the promotion of its tensile strength. Via shocking and soaking in deionized water, the PVA residue was removed from the PDV composites, which can avoid affecting subsequent experiments. The chemical structure after the removal of PVA from PDV composites was tested using FTIR to verify the complete removal of PVA from DHVs. Herein, PDV with improved processability provided theoretical and technical support for the subsequent development of high-performance decellularized biological valves.

## 2. Materials and Methods

### 2.1. Materials

Powder PVA (MW: 7.2–8.14 × 10^4^ g/mol) was purchased from Yingjia Industrial Development Co., Shanghai, China. Cell counting kit-8 (CCK-8) was obtained from Biosharp, Hefei, China. Phosphate buffered saline (PBS), Penicillin/streptavidin (PS), and Trypsin were purchased from HyClone, South Logan, UT, USA. 4’,6-diamidino-2-phenylindole (DAPI) fluorescent staining solution, Calcein-AM/PI Live Dead Cell Assay Kit, TRITC-Phalloidin and Tris(hydroxymethyl)aminomethane hydrochloride (TRIS-HCI) Buffer were purchased from Biyuntian Biotechnology Co., Shanghai, China. TritonX-100 solution, 3-[(3-Cholamidopropyl)-dimethyl-ammonio]-1-propane sulfonate (CHAPS), Tributyl phosphate, N-Decyl-N, N-dimethyl-3-ammonio-1-propanesulfonate (SB3-10), Amidosulfobetaine-14 (ASB-14) and Benzonase nuclease were obtained from Sigma-Aldrich, St. Louis, MO, USA.

### 2.2. Preparation of Decellularized Valves

Fresh pig hearts taken from the slaughterhouse (Jiangxia Foreign Trade Hengxing Mechanized Slaughtering Plant, which is a licensed abattoir producing meat for consumption operating under strict government protocols. The fresh pig hearts were waste products; therefore, the ethical approval of fresh pig hearts was not required.) were placed in normal saline containing heparin sodium and quickly transferred to the laboratory. The porcine aortic valves (length averages 2.0 cm, width averages 2.0 cm) were cut out under aseptic conditions, shaken, and rinsed in PBS until the surface of the valves was free of blood and then removed. The valves were placed in antibiotic-containing PBS at 4 °C for 12 h.

The valves were then decellularized on the surface using water-soluble and lipid-soluble proteins, and the valves were placed in a six-well plate with three parallels. The specific steps were as follows: (1) Porcine aortic heart valves were placed in a concentration of 2% CHAPS and 2 mmol/L tributyl phosphate in TRIS-HCI buffer (pH = 7.8) at room temperature and shaken by shaker for 24 h. (2) Rinse six times using deionized water, requiring more than 10 min each time. They were placed in TRIS-HCI buffer containing 2% CHAPS, 2 mmol/L tributyl phosphate, 1% ASB-14, and 2% SB3-10 (pH = 7.8) and continued shaking at room temperature for 24 h. (3) They were removed and rinsed four times with PBS for 6 h each time. They were then placed in TRIS-HCl buffer containing 1 mmol/L MgCl_2_ and 100 units/mL Benzonase (pH = 8), and shaking was continued for 24 h at 37 °C. (4) The valves were rinsed more than four times for 6 h each time using PBS. The purpose of this rinsing was to remove the reagents and nuclei remaining on the surface of the valves and, finally to obtain decellularized valves. The decellularized valves were placed in PBS containing antibiotics and stored at 4 °C.

### 2.3. Preparation of Polyvinyl Alcohol (PVA) Solution [32]

The PVA powder and deionized water were mixed at the mass ratios of 10%, 12%, 14%, and 16%. Then, the mixture was stirred at 200–800 rpm/min at 50–90 °C for 1–3 h. After complete dissolution, a homogeneous and transparent PVA solution was obtained. After stirring, the PVA solution was defoamed under a vacuum of −0.1 to −0.05 MPa for 2 to 6 h to obtain uniform PVA solutions with mass ratios of 10%, 12%, 14%, and 16%.

### 2.4. Preparation of PVA/Decellularized Valves Composite Membranes

The water on the surface of decellularized valves was removed and absorbed with filter paper. Then, decellularized valves were placed in a Petri dish and spread out. The PVA solution made in the previous step was evenly spread in the Petri dish, then the Petri dish was dried in an oven at 50 °C for 6–8 h. The PVA/decellularized flap composite membrane was obtained.

After the dehydration of the decellularized valve membranes, the PVD composite membranes were transparent. The prepared composite membranes were soaked in deionized water to remove PVA from the surface of the decellularized valves, and the decellularized valves obtained after treatment with different concentrations of PVA solution were placed in PBS containing antibiotics and stored at 4 °C.

### 2.5. H&E Staining

Tissues were immersed in 4% paraformaldehyde overnight. After overnight immersion, the tissues are dehydrated in gradient alcohol, and the tissue blocks must be transparent after alcohol dehydration. Then, the tissues were embedded in paraffin blocks for sectioning. H&E staining of tissue sections was performed.

### 2.6. Yarn Sutured Decellularized Heart Valves

The decellularized heart valves were put into a 6-well plate and poured into a 12% PVA solution. After standing for half an hour to defoam, a 6-well plate was put into the oven to make PVA into a film, then the composites PVD were formed. The PVD composites were aligned with the calibration paper and sutured using Polylactic acid (PLA) yarns. The PLA yarns were spaced 2, 3, and 4 mm. After suturing, the sutured composite valves with the calibration paper were soaked in deionized water to dissolve the PVA.

### 2.7. Surface Morphology Characterization

The decellularized valves with the PVA removed were taken from the PBS containing antibiotics, of which the surface water was absorbed by filter paper. The decellularized valves were laid flat in a Petri dish and placed in a −20 °C refrigerator for 10 h. The Petri dish was then placed in a −80 °C. The samples were then lyophilized in a freeze dryer at −80 °C and 5 Pa pressure. The lyophilized samples were removed and set aside. The samples were cut into small pieces with a razor blade and attached to the electron microscope stage using conductive adhesive. The samples were observed microscopically using a JSM-6700F scanning microscope (Nippon Electron, Tokyo, Japan). The sample was sprayed with gold for 120 s and then placed under the scanning microscope.

### 2.8. Flexibility Characterization

The decellularized valves with the PVA removed were taken from the PBS containing antibiotics and kept moist. With the parallel clamps, the wetted valve was held at 5 mm in a direction parallel to the longitudinal direction of the valve. The sample was clamped to remain in a natural drape. The camera was fixed at the same position and angle to take pictures. Five samples from each group were prepared for testing. The angle of the valve overhang was then calculated using Image J 1.52a image analysis software (National Institutes of Health, Bethesda, MD, USA). The angle was calculated using the horizontal line, the intersection of the sample and the test tube clamp, and the angle formed by the sample.

### 2.9. Mechanical Properties Characterization

The decellularized valves with the PVA removed were taken from the PBS containing antibiotics. The valve was cut into several pieces with a width of 5 mm along the longitudinal direction for the mechanical test. The universal testing machine (Instron 5943, Norwood, MA, USA) was used with a 10 N sensor and a 50 N fixture and the fixture spacing was set at 8 mm, and the fixing speed was 5 mm/min for tensile testing (n = 5, 4.5 × 10 mm).

To make the clamp hold the sample better, the cut sample was laid flat on the filter paper. Meanwhile, the filter paper/sample was cut, and the filter paper/sample was held flat between the clamps with tweezers. After the clamp was held, the filter paper was wetted. Finally, the filter paper was removed from the sample with tweezers to avoid interference of the filter paper with the mechanical properties of the decellularized valves.

### 2.10. Fourier Transform Infrared Spectrometer (FTIR) Analysis

The decellularized valves with the PVA removed were taken from the PBS containing antibiotics, of which the surface water was absorbed by filter paper. The decellularized valves were laid flat in a Petri dish and placed in a −20 °C refrigerator for 10 h. The Petri dish was then placed at −80 °C. The samples were then lyophilized in a freeze dryer at −80 °C and 5 Pa pressure. The lyophilized samples were removed and set aside. FTIR was performed using Tensor-27 Fourier transform infrared spectrometer (Bruker, Saarbrucken, Germany). The test parameters were a scanning range of 4000–600 cm^−1^, resolution of 8 cm^−1^, scanning number of 64, test temperature at room temperature, and attenuating total reflection.

### 2.11. Sterilization of Samples

The decellularized valves with the PVA removed were sterilized. Then, each sample was cut into two using a razor blade. The valves were sterilized by shaking for 3 h at room temperature in 0.1% peroxyacetic acid disinfection solution at 100 rpm/min speed of the shaker. The sterilized samples were then rinsed in PBS for 3 h at room temperature with a solution change every hour.

### 2.12. Cell Culture

Human umbilical vein endothelial cells (HUVECs) were purchased from Nanjing Keygen Biotech Co., Ltd. (KG110, Nanjing, China). HUVECs were cultured in this study. After heating the water bath to 37 °C, the lyophilized tubes containing HUVECs were removed from the liquid nitrogen tank at −196 °C, placed in the water bath, and stirred rapidly until the cryopreservation solution melted. After the cell cryopreservation solution was completely dissolved, the cells were quickly aspirated and transferred to a centrifuge tube containing 3 mL of culture medium, blown and homogenized, and set aside. The medium consisted of 89% high-sugar Dulbecco’s modified eagle medium (DMEM) cell medium, 10% Fetal Bovine Serum (FBS), and 1% penicillin/streptomycin. The tubes were centrifuged at 1000 rpm/5 min at 22.5 °C, then the supernatant was discarded, and the cell precipitate was left. The appropriate amount of fresh medium was added to the centrifuge tube. Cells were blown down from the wall of the centrifuge tubes and dispersed evenly. Cell suspension was dispensed into cell culture flasks at a ratio of 1:3 and incubated in a cell culture incubator (37 °C, 5% CO_2_).

When the cell density reached 80%–90% in the culture flask, the cell passaging operation was performed. The culture medium was aspirated from the flask. The cells were rinsed twice with PBS so that the residual medium in the culture flask was completely cleaned. An appropriate amount of trypsin at 0.25% concentration was pipetted into the culture flask just to cover the bottom of the flask and placed in the incubator for 3 min to allow for digestion. An inverted microscope observed the cells, and the digestion reaction was terminated by adding an equal volume of medium when the cells appeared to shrink into a spherical shape. The cells were repeatedly blown from the bottom of the flask using a pipette gun, aspirated into a centrifuge tube, and then centrifuged in a centrifuge. After centrifugation, the cells were transferred into new culture flasks at a ratio of 1:4 and incubated in a cell culture incubator (37 °C, 5% CO_2_). Cells were passed for 3–5 generations in the same manner as above and set aside.

### 2.13. Cell Adhesion

After treatment with different concentrations of PVA and removal, HUVECs were cocultured with the decellularized valves by direct contact. Sterilized samples were fixed in 48-well plates, followed by injection of 200 µL of cell suspension at a density of 2 × 10^5^ cells/mL onto each sample (n = 4, d = 11 mm) and supplemented with sufficient cell culture medium for 1, 2, 3 and 6 h. After incubation, the original medium was aspirated. The well plates were rinsed three times with PBS.

Fresh medium and CCK-8 were added to the well plate and incubated in the cell incubator for 2 h. After 2 h, the well plate was taken out. Suspension was aspirated from each well and placed in a 96-well plate. The optical density (OD) value of the suspension in each well was measured at 450 nm using an enzyme marker. The adhesion rates were calculated as: Cell adhesion rate (%) = (OD_experimental_ − OD_blank_)/(OD_control_ − OD_blank_) (Blank group: OD value with medium, CCK-8 solution; Control group: OD value with HUVECs, medium, CCK-8 solution and the HUVECs were seeded at the 96-well plate directly).

### 2.14. Cell Proliferation

Sterilized samples were fixed in 48-well plates, followed by injection of 200 µL cell suspension at a density of 2 × 10^5^ cells/mL onto each sample (n = 4, d = 11 mm) and supplemented with sufficient cell culture medium for 1, 3, 5 and 7 d. After HUVECs cocultured with the different decellularized valves by direct contact, the original medium was aspirated. The well plates were rinsed three times with PBS. Fresh medium and 20 µL CCK-8 were added to the well plate and incubated in the cell incubator for 2 h. After 2 h, the well plate was taken out. Suspension was aspirated from each well and placed in a 96-well plate. The OD value of the suspension in each well was measured at 450 nm using an enzyme marker.

### 2.15. Cell Morphology

To visualize the morphology of HUVECs clearly, TRITC-Phalloidin stained cytoskeleton and DAPI stained nucleus.

Sterilized samples (n = 2, d = 11 mm) were fixed in 48-well plates, and 200 µL cell suspension was then inoculated onto each sample at a density of 1 × 10^5^ cells/mL. After incubation of 24 h by direct contact, the culture medium was aspirated from the surface of the valves, washed twice with PBS, and 200 µL 4% paraformaldehyde was added to each well for 20 min at room temperature to fix the cells.

After removing paraformaldehyde, the wells were washed twice with PBS, and 200 µL 0.3% TritonX-100 solution was added to the wells and permeabilized for 10 min at room temperature. After removing the TritonX-100 solution, the plates were washed twice with PBS, and 200 µL of TRITC-Phalloidin was added to each well and incubated for 30 min in a cell incubator protected from light.

The TRITC-Phalloidin was aspirated from the well, which was rinsed twice with PBS. DAPI dye was added to the wells and reacted at room temperature for 5 min. The DAPI dye was aspirated from the well, which was rinsed twice with PBS. The cytoskeleton (red fluorescence) and nucleus (blue fluorescence) were observed by photographing with a cell imaging system.

### 2.16. Statistical Analysis

In the study, the data were analyzed using GraphPad Prism 8.3 software (GraphPad Software, San Diego, CA, USA). The mean values ± standard deviation (SD, n ≥ 3) were used to represent all data. The level of significant difference was determined using a two-tailed Student’s test (* *p* < 0.05, ** *p* < 0.01) unless otherwise stated.

## 3. Results

### 3.1. Histological Staining of Decellularized Valves Treated with PVA Solution

**Figure 2:**
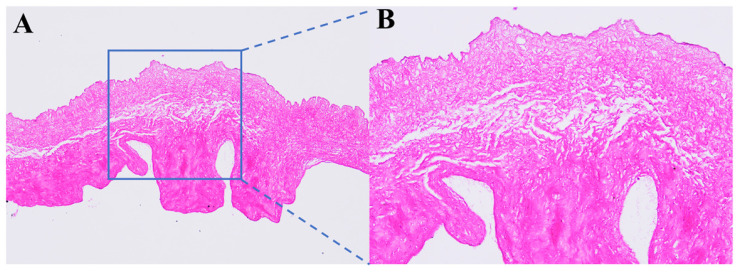
The H&E staining of decellularized valves treated with 12% PVA cross-section under the magnification of 100× (**A**) and 200× (**B**).

After a series of decellularization processes, decellularized valves were successfully prepared in this study, and the effect of decellularization was further evaluated. The results of the H&E staining analysis (Figure 2) further confirmed that the nucleus of the cell was eliminated after the decellularization procedure. In future clinical translation, decellularization will help to reduce immunological rejection following xenotransplantation. In addition, the extracellular matrix fibers can be seen in the H&E staining of the decellularized valve treated with a 12% PVA cross-section. Therefore, the H&E staining of the decellularized valve treated with a 12% PVA cross-section can show the valve’s physiologic architecture is retained.

### 3.2. Microscopic Morphology of Decellularized Valves Treated with Different Concentrations of PVA

**Figure 3:**
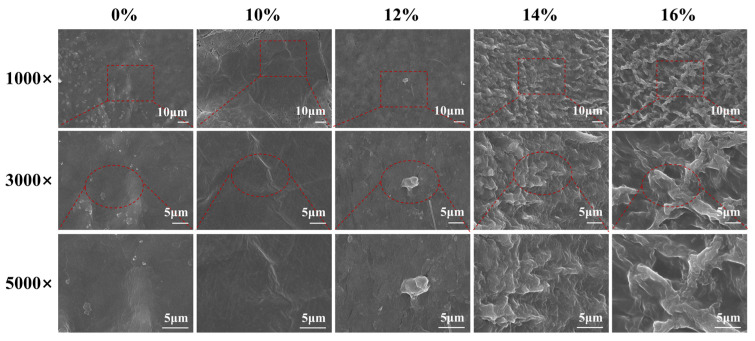
The microscopic morphology characterizations of decellularized valves treated with different concentrations of PVA. Representative SEM images of decellularized valves treated with different concentrations of PVA (0%, 10%, 12%, 14%, and 16%) under the magnification of 1000×, 3000×, and 5000×. Red dotted circle and box show the local of low magnification SEM images.

In this study, the PVA solution was fixed on the surface of the decellularized valve as a template. Then, the PVA/decellularized porcine valves (PDV) composite was synthesized, which could be easily held for further processing. Subsequently, the PVA of decellularized valves was removed to avoid changes in the morphology of the valves. Finally, the decellularized porcine valves were obtained after PVA treatment and PVA removal. The microscopic morphology of the decellularized porcine valves treated with different PVA concentrations was observed using scanning electron microscope (SEM). The SEM images showed (Figure 3) that the decellularized valve without PVA treatment (0% PVA) had grooves on the surface at 1000× magnification. With the corresponding enlarged SEM images (3000× and 5000× magnified), the surface morphology was observed to be relatively smooth. The results of decellularized valves with 10% and 12% PVA treatment were also consistent with that of decellularized valves without PVA treatment, which the reason might be that the decellularized valves with 10% and 12% PVA treatment completely removed the PVA residues from the surface during the following process. In contrast, the microscopic morphologies of decellularized valves with 14% and 16% PVA treatment were observed to be uneven at 1000× magnifications. The results showed the microscopic morphology of the decellularized valves changed after 14% and 16% PVA treatment, which might be caused by two factors. Firstly, the PVA residue on the surface of decellularized valves with a high concentration of PVA treatment could not be removed completely, showing uneven morphology. Secondly, the water in the oven with the high concentration of PVA was more difficult to remove than that of a low concentration of PVA. The time of decellularized valves in the oven was longer, leading to the change of surface morphology of the decellularized valves under the effect of high temperature. Therefore, the microscopic morphology of decellularized valves with 14% and 16% PVA treatment changed. 

### 3.3. FTIR Analysis

**Figure 4:**
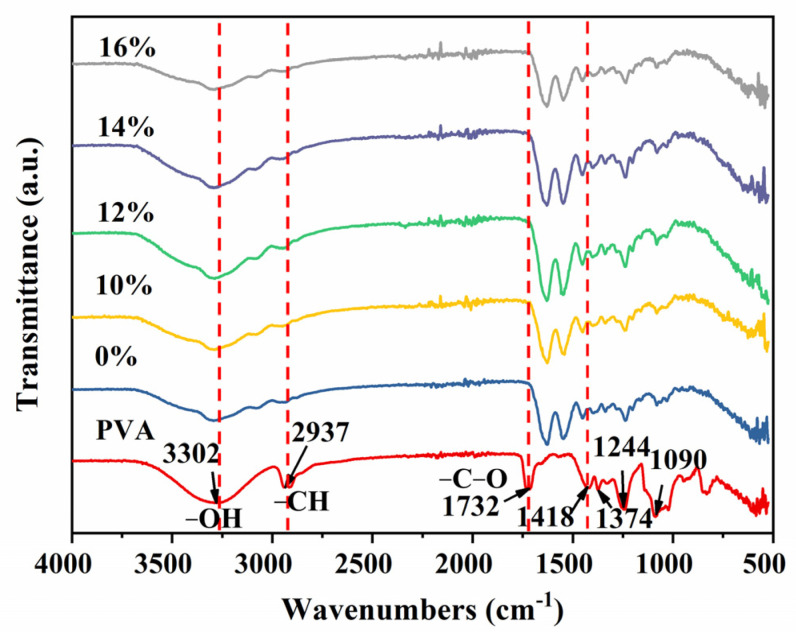
The FTIR spectra of decellularized valves treated with different concentrations of PVA. Red dotted lines show corresponding characteristic peaks.

Further, to verify the removal of PVA from decellularized valves, the decellularized valves treated with different PVA concentrations were subsequently analyzed using FTIR spectroscopy, and pure PVA was used as control (Figure 4). The results showed the infrared spectra of decellularized porcine valves after different concentrations of PVA treatment and PVA removed. Typical bands for vinyl polymers were exhibited by pure PVA [33]. It could be seen that 3302 cm^−1^ and 2937 cm^−1^ were the stretching vibration peaks of O-H and C-H asymmetric stretching vibration peaks of PVA, respectively. The peak at 1732 cm^−1^ was attributed to the stretching of C-O. The bands observed at around 3300 and 2926 cm^−1^ correspond to O-H group stretching and C-H stretching. The signals of the C-O bonds, the C-H bending, the O-H in-plane bending, and the C-O stretching vibrations can be seen in the figure [34,35]. The peak at 1432 cm^−1^ was attributed to the symmetric bending of C-H. Among them, 2937 and 1732 cm^−1^ were characteristic peaks of PVA. Additionally, it could be clearly seen that the 10%, 12%, 14%, and 16% PVA-treated decellularized valves did not overlap at all with the peaks of pure PVA, which were consistent with the untreated decellularized valves. The results indicated there was no PVA left on the surface of the decellularized valve after the PVA film was removed from the surface by deionized water. Therefore, with the SEM images of decellularized valves treated with different concentrations of PVA, the changes in the microscopic morphology of the 14% and 16% PDV composites were not residual PVA, but the result of the longer dehydration time of the higher concentration of PVA than that of lower concentration of PVA with high temperature.

### 3.4. Comparison of Flexibility of Decellularized Valves Treated with Different Concentrations of PVA

**Figure 5:**
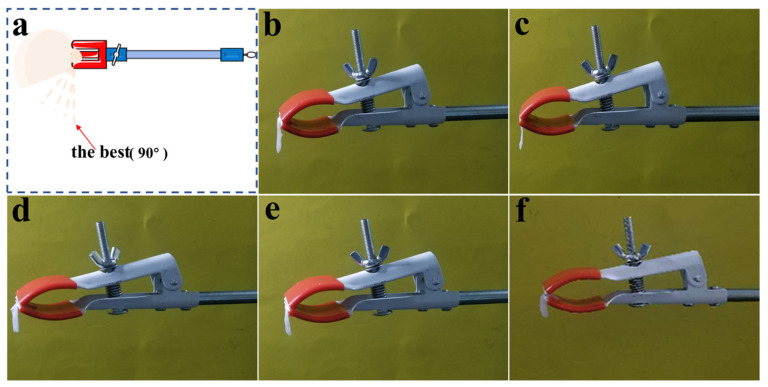
Evaluations on the flexibility of decellularized valves treated with different concentrations of PVA solution. Schematic illustration of test of bending degree (**a**); bending degrees of decellularized valve treated with 10% (**b**), 12% (**c**), 14% (**d**), and 16% (**e**) PVA solution and pure decellularized valve (**f**) after contacting with water.

According to previous studies, decellularized valves could not maintain the overhanging state in natural wetting conditions after modification and cross-linking treatment, which reflects the modulus of valves [36]. To investigate the flexibility of the PDV composites after removing PVA, bending degrees in a wet state were measured after contact with water, and the pure decellularized valve was used as control (Figure 5). The results showed the natural drop state of the decellularized valves with 10%, 12%, 14%, and 16% PVA treatment, and pure decellularized valves were all naturally vertical downward in a wet state. In contrast, the pure decellularized valves had a fuller morphology and higher water content compared with those in other groups, indicating the flexibility of the valve did not change after different concentrations of PVA treatment.

### 3.5. Comparison of Mechanical Properties of Decellularized Valves Treated with Different Concentrations of PVA

**Figure 6:**
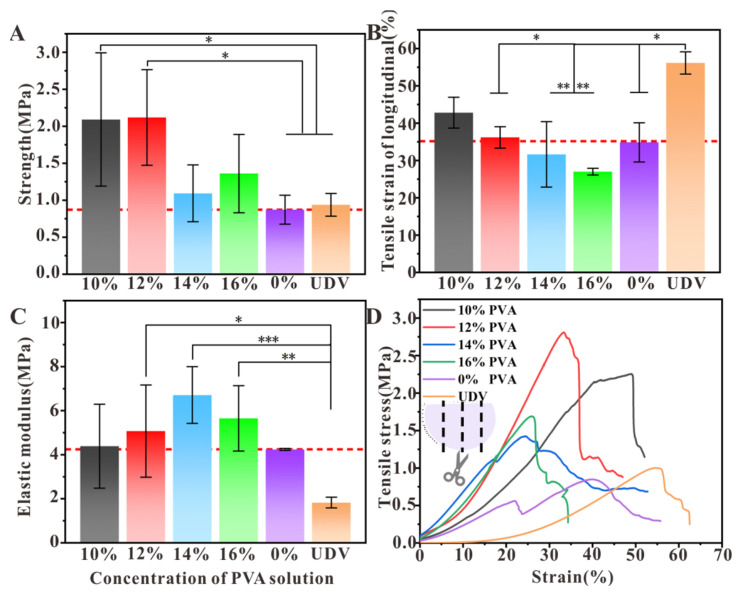
The mechanical characterizations of decellularized valves treated with different concentrations of PVA solution: Strength (**A**), Tensile strain of longitudinal (**B**), Elastic modulus (**C**), and Stress–strain curve (**D**). (UDV: un-decellularized valves; red dotted line: the reference line; Error bars: ±SD, * *p* < 0.05, ** *p* < 0.01, *** *p* < 0.001).

To explore mechanical properties, the changes of strength, the tensile strain of longitudinal, elastic modulus, and stress–strain curve of decellularized valves after PVA solution treatment and un-decellularized valves were measured. The tensile testing for the different valves was further carried out, as presented in Figure 6. The decellularized valves treated with 0%, 10%, 12%, 14%, 16% PVA, and un-decellularized valves showed a tensile strength of 0.87, 2.09, 2.12, 1.09, 1.36, and 0.94 MPa, respectively, and the elastic modulus was 4.24, 4.39, 5.07, 6.71, 5.65, and 1.83 MPa, respectively. The decellularized valves treated with the PVA concentration (12%) exhibited a higher tensile strength (2.12 ± 0.5 MPa) than the decellularized valves (0.87 ± 0.3 MPa) and un-decellularized valves (0.94 ± 0.2 MPa), indicating there was a difference in the strength of decellularized valves before and after different concentrations of PVA treatment (Figure 6A). For tensile strain of longitudinal, the un-decellularized valves showed higher strain than decellularized valves, and the 10%, 12% PVA-treated decellularized valves showed higher elastic deformation than the untreated decellularized valves, and there was a gradual decrease in regularity (Figure 6B). Therefore, the treatment with PVA can improve the valves’ tensile strain. The result of elastic modulus showed a slight increase after treatment with 10%, 12%, and 14% PVA with a tendency to increase and then decrease. The greater the modulus of elasticity, the greater the ability of the material to resist deformation. When the concentration reached 16%, the decellularized valves exhibited hardness and lack of elasticity, which led to the modulus of the decellularized valves decreasing (Figure 6C). Additionally, the results of the stress–strain curve showed the decellularized valves treated with PVA led to higher tensile stress and strain than the untreated decellularized valves, and the longitudinal stress was about 2.72 MPa after 12% PVA-treated decellularized valves. In summary, we found that the different concentrations of PVA treatment in the system resulted in improved tensile strength. These results confirmed the successful synthesis of the tough and flexible decellularized valves utilizing PVA as a dynamic template.

### 3.6. Cytocompatibility of Decellularized Valves Treated with Different Concentrations of PVA

**Figure 7:**
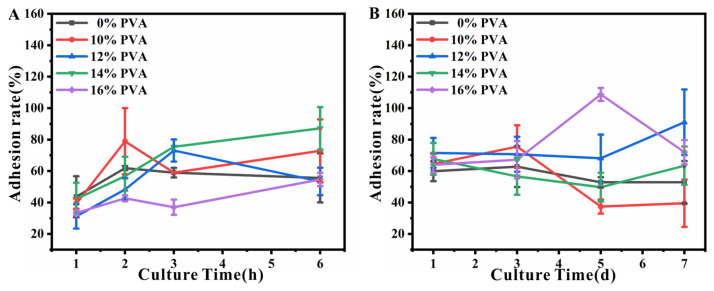
Results of in vitro cell adhesion. The HUVECs adhesion rates of decellularized valve treated with different concentrations of PVA solution at 1, 2, 3, and 6 h (**A**); 1, 3, 5, and 7 d (**B**).

Next, the cytocompatibility of decellularized valves treated with different concentrations of PVA was calculated using cell adhesion rates at different times (Figure 7). After HUVECs incubated with decellularized valves treated with different concentrations of PVA for 1, 2, 3, and 6 h, cell adhesion rates on the surface of decellularized valves were calculated (Figure 7A), which reflected the speed of cell adhesion. The results showed the cell growth of the decellularized valves without PVA treatment, and the decellularized valves with different concentrations of PVA treatment maintained stable adhesion rates, and the difference in rates was not significant. Therefore, PVA treatment did not affect the cell adhesion performance of decellularized valves. Further, HUVECs’ adhesion rates of decellularized valves with different concentrations of PVA at 1, 3, 5, and 7 d were calculated (Figure 7B), which reflected the rate of cell proliferation. As shown in Figure 7B, with the concentration of the PVA up to 12%, the adhesion rate was higher than 91% after 7 days’ incubation. The results showed the cell growth of the decellularized valves with or without PVA treatment maintained a stable growth rate. Therefore, PVA treatment did not cause cytotoxicity.

**Figure 8:**
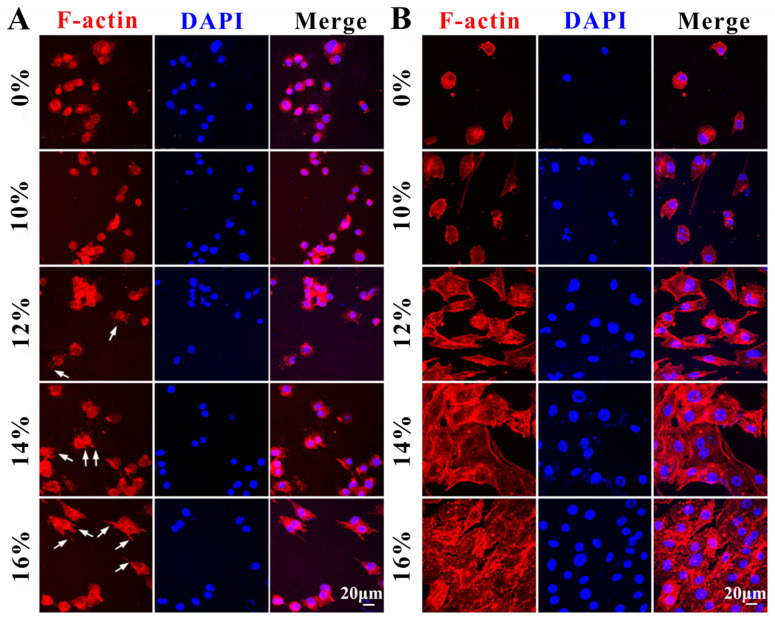
The in vitro characterizations of cell morphology. TRITC-phalloidin (red) and DAPI (blue) staining fluorescence images of HUVECs morphology on the surface of decellularized valves treated with different concentrations of PVA solution after 24 h culture (**A**) and 7 d culture (**B**). (White arrows: cells’ pseudopods).

According to previous reports, the adhesion, proliferation, and differentiation of cells on the surface of the material are needed to seek an appropriate position with optimal cytocompatibility [37,38]. To investigate cell morphology on the surface of decellularized valves treated with different concentrations of PVA after 24 h and 7 d, TRITC-Phalloidin (red) and DAPI (blue) fluorescence were used to observe cytoskeleton and cell nucleus (Figure 8), respectively. As shown in Figure 8A, the morphology of cells on the surface of the decellularized valves treated with 0% and 10% PVA cultured for 24 h were scattered but not completely spread out. While red and blue fluorescence on the surface of the decellularized valves treated with 12%, 14%, and 16% PVA were completely spread out, indicating the cells formed pseudopods (white arrows) and polygonal shapes with a clear tendency to spread in all directions. Furthermore, the cytocompatibility of decellularized valves treated with different concentrations of PVA was evaluated by cell proliferation after cultured for 7 d (Figure 8B). As observed, the cells on the surface of the decellularized valves treated with 0% and 10% PVA solutions had an oval cell shape and did not spread out completely, indicating the adhesion of the valves to the cells was low. The cells on the surface of the decellularized valves after 12%, 14%, and 16% PVA treatment showed the same morphology as the 1st day, with the cells spreading in all directions, exhibiting a polygonal cell shape and good adhesion properties to the surface of decellularized valves. In particular, when the PVA concentration were 14% and 16%, the surface of the decellularized valves were almost completely covered by HUVECs, which verified excellent cytocompatibility. The results might be due to the longer dehydration time of the higher concentration of PVA than that of lower concentration of PVA with high temperature, making the changes in the surface morphology of the 12%, 14%, and 16% PDV composites, which supported the adhesion of cells on the surface.

### 3.7. Processability of Decellularized Valves Treated with Different Concentrations of PVA—An In Vitro Study

**Figure 9:**
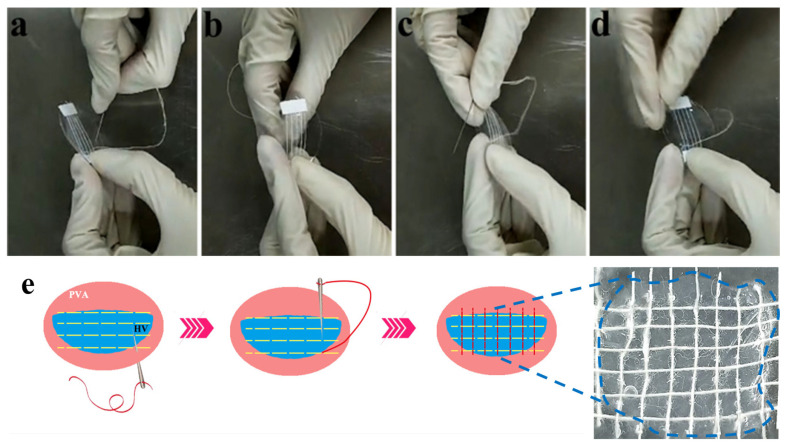
Suture procedure of decellularized valves treated with 12% PVA. Digital photographs of suturing PDV with PLA yarn (**a**–**d**); schematic illustration of suture procedure (**e**).

To explore the processability of decellularized valves treated with 12% PVA, they were sutured with PLA yarn (Figure 9). The result showed that decellularized valves treated with 12% PVA could be held and repeatedly punctured and sutured with stitches without fragmenting the valve, indicating the decellularized valves treated with 12% PVA could be applied for further processing applications.

## 4. Discussion

Currently, tissue engineering of heart valves using decellularized valves has been a promising approach to meet the replacement, regeneration, and growth needs of heart valves. Previous studies have shown that decellularized valves reduce immunogenicity and the risk of unknown infections while being less susceptible to calcification [39]. Here, we utilized PVA and decellularized valves to address the issue that they are difficult to apply in the clinic. The PDV composite membranes were formed by laminating PVA with decellularized valves to ease the application of the decellularized valves. The effect of PVA concentration on the microscopic morphology, softness, mechanical properties, and biocompatibility of decellularized valves was investigated.

Whether the PDV composite membranes could be used to construct tissue engineering of heart valves was the next challenge. The strength and modulus of the valve after decellularization have decreased to a certain extent, so its mechanical properties need to be improved to ensure smooth subsequent processing. Processability is an essential indicator in the subsequent preparation process. Thus, this study investigated the improved processing properties of decellularized valves treated with different concentrations of PVA.

The decellularized valves were treated using PVA due to their flexibility and inertness, which aims to minimize any adverse effects on the valves in this study. After determining the optimal PVA concentration, there were no chemical reactions with the valves, and only physical fixation was used to enhance the suture resolution. Therefore, the purpose of PVA fixation is to increase the possibilities for suturing, with a focus on fiber-reinforced processes. In conclusion, PVA, as a template technology with unique advantages such as low cost, simplicity of operation, and the ability to control various geometries, has been widely utilized in improving mechanical properties [40].

The natural drop state refers to the decellularized valves being almost perpendicular to the clamps. Our results show that both the treated decellularized valves and the original decellularized valves are in the natural downward vertical state. The flexibility of the decellularized valve did not change after treatment with different concentrations of PVA solution and remained consistent with the untreated decellularized valve.

Our mechanical and suture results further indicated the mechanical properties of PVA-treated decellularized valves were somewhat improved, thereby providing better initial processability and mechanical support, which was useful for surgical procedures [41]. The decellularized valve treated with 14% PVA shows statistically significant differences in elastic modulus and tensile strain from the control decellularized tissue.

To establish a relationship between the cytocompatibility and the decellularized valves treated with PVA, HUVECs were seeded on the decellularized valves, and the cell adhesion, proliferation, viability, and morphology of the decellularized valves were evaluated. Before coculturing HUVECs by direct contact with the decellularized valves, the decellularized valves were treated with different concentrations of PVA and then removed PVA. The white arrows indicate cells formed pseudopods and polygonal shapes with a clear tendency to spread in all directions in Figure 8. Previous research has demonstrated the favorable biocompatibility of PVA in both in vitro and in vivo tests [42,43,44]. The in vitro data presented in this study support the cytocompatibility of decellularized valves treated with PVA. As a result, the decellularized valves treated with different concentrations of PVA kept cytocompatibility.

This study demonstrates that the PDV not only enhances the processability of decellularized valves but also exhibits cytocompatibility in vitro. While there are still unanswered questions regarding certain aspects, such as the absence of an in vivo investigation in animal models and the specific mechanisms involved, such as platelet adhesion or the release of functional factors, the present study has introduced an innovative approach to tissue engineering of heart valves.

## 5. Conclusions

In this study, the effect on decellularized valves treated with different concentrations of PVA was investigated by changing the concentration of PVA. Owing to the template of PVA, the resulting decellularized valves showed good mechanical strength, which could overcome the problem of limiting the processability of the decellularized valves. The good water-solubility of PVA enables PDV composites to be easily processable application in the TEHV. Notably, the valves are treated with the help of the flexibility and inertness of PVA to increase the suture resolution of the valves only through physical fixation and minimize its effect on the valves. Compared with 14% and 16% PVA, the microscopic morphology of the decellularized valve treated with 12% PVA kept intact. Overall, 12% of PVA has verified the ability to improve processability. After yarn enhancement, PVA will be removed. The yarn-enhanced valves will be easier to suture at the time of implantation because the presence of circumferential yarns will increase their suture strength retention. Therefore, this study presented a meaningful strategy for improving the cytocompatibility and processability of decellularized tissue, which largely facilitates its application and prevents local damage.

## Data Availability

The data that support the findings of this study are available from the corresponding author upon reasonable request.
